# Developmental programming of hypothalamic neuronal circuits: impact on energy balance control

**DOI:** 10.3389/fnins.2015.00126

**Published:** 2015-04-21

**Authors:** Thanuja Gali Ramamoorthy, Ghazala Begum, Erika Harno, Anne White

**Affiliations:** ^1^Faculty of Life Sciences, University of ManchesterManchester, UK; ^2^School of Clinical and Experimental Medicine, University of BirminghamBirmingham, UK; ^3^Faculty of Medical and Human Sciences, Centre for Endocrinology and Diabetes, University of ManchesterManchester, UK

**Keywords:** fetal programming, pro-opiomelanocortin, glucocorticoid receptor, epigenetics, hypothalamus

## Abstract

The prevalence of obesity in adults and children has increased globally at an alarming rate. Mounting evidence from both epidemiological studies and animal models indicates that adult obesity and associated metabolic disorders can be programmed by intrauterine and early postnatal environment- a phenomenon known as “fetal programming of adult disease.” Data from nutritional intervention studies in animals including maternal under- and over-nutrition support the developmental origins of obesity and metabolic syndrome. The hypothalamic neuronal circuits located in the arcuate nucleus controlling appetite and energy expenditure are set early in life and are perturbed by maternal nutritional insults. In this review, we focus on the effects of maternal nutrition in programming permanent changes in these hypothalamic circuits, with experimental evidence from animal models of maternal under- and over-nutrition. We discuss the epigenetic modifications which regulate hypothalamic gene expression as potential molecular mechanisms linking maternal diet during pregnancy to the offspring's risk of obesity at a later age. Understanding these mechanisms in key metabolic genes may provide insights into the development of preventative intervention strategies.

## Introduction

The number of reported cases of obesity has risen dramatically in the developed world and is one of the greatest public health challenges we face due to the projected burden on future health care services. Current estimates suggest that more than a third of adults in the United States are obese and according to the Department of Health in the UK, more than half of the adult population is either overweight or obese (Department of Health, 2014). Obesity is also a risk factor for a number of other associated metabolic disorders such as type 2 diabetes, cardiovascular disease, hypertension, stroke, and osteoarthritis (Abbasi et al., 2002; Steinberger et al., 2003; Qatanani and Lazar, 2007; Chiarelli and Marcovecchio, 2008). It is undoubtedly multifactorial, occurring as a result of both complex genetic traits and environmental factors.

The gene-environment interaction represents a likely factor in the recent demographic shift toward a more obese phenotype. This comes as growing evidence shows that fetal and early postnatal environments influence the risk of developing obesity. Epidemiological and animal studies show that nutritional imbalances such as under- and over-nutrition during critical ontogenic periods induce persistent physiological alterations. These changes are strongly associated with metabolic disorders such as obesity, type 2 diabetes, and cardiovascular disease in adult offspring (Blackmore and Ozanne, 2013; Duque-Guimaraes and Ozanne, 2013; Hanson and Gluckman, 2014).

Programming of metabolic disorders in humans by intrauterine experiences was first explored by Barker et al. (1990). They observed a strong association between low birth weight and the risk of cardiovascular disease and type 2 diabetes in adulthood (Barker et al., 1990, 1993; Hales et al., 1991). Other retrospective analyses have also demonstrated that early life experiences can increase the susceptibility of developing metabolic disorders in adulthood (Ravelli et al., 1976, 1999). These observations led to the fetal programming hypothesis also known as “Developmental origins of health and disease (DOHaD)” or “thrifty phenotype” hypothesis. This theory suggests that the fetus will adapt to adverse changes in the intrauterine environment such as maternal undernutrition. However, increased nutritional availability in postnatal life can result in the development of metabolic syndrome (Hales and Barker, 2001). Emerging evidence confirms that maternal overnutrition during pregnancy and increased birth weight also increase the incidence of obesity in the offspring. Thus, the relationship between human birth weight and the potential of developing adult obesity and other metabolic disorders is a “U-shaped” curve with risk from both maternal under- and over-nutrition (Curhan et al., 1996; Pettitt and Jovanovic, 2001; Ong, 2006).

Importantly, fetal programming by both maternal under- and over-nutrition involves perturbations in the hypothalamic appetite regulatory systems which are established during early development (McMillen et al., 2005; Bouret, 2009; Stevens et al., 2011; Ross and Desai, 2014). The hypothalamus controls food intake and energy expenditure and is a prime target of developmental programming by maternal nutritional imbalance that predisposes the offspring to permanent risks of metabolic disorders. Epigenetic alterations of key metabolic genes in the hypothalamus are central in regulating this process. The purpose of this review is to discuss evidence from recent studies supporting hypothalamic programming of obesity and the role of epigenetic gene regulation in mediating phenotypic alterations in the offspring as a result of a maternal nutritional insult.

## Hypothalamic neuronal circuits controlling energy balance

Energy balance is controlled centrally, predominantly by the hypothalamus. The principal area containing these circuits is the arcuate nucleus (ARC), but within the hypothalamus the paraventricular nucleus (PVN) and the dorsomedial hypothalamus (DMH) also have a role in the regulation of energy balance. The ARC is known as the “feeding” center of the brain. It is a region close to the 3rd ventricle and the median eminence. This region of the brain has a “leaky” blood brain barrier, which allows the access of peripheral hormones to this part of the brain. The ARC contains both anorexigenic and orexigenic neurons. Together, the peptides released from these two sets of neurons counterbalance each other to control food intake and energy expenditure and ultimately have a major role in the control of body weight.

### Anorexigenic ARC neurons

**Figure 1:**
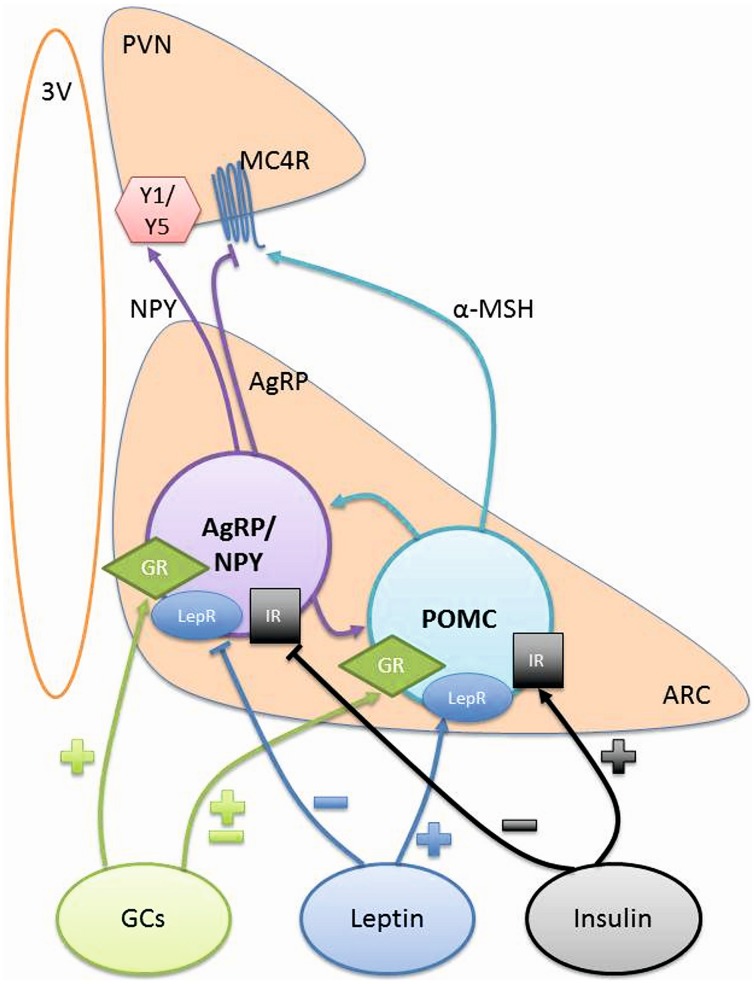
**Hypothalamic signaling controlling energy balance**. POMC and AgRP/NPY neurons project from the ARC to the PVN to control food intake and energy balance. The signaling from these neurons can be influenced by GCs, Leptin, and Insulin. ARC, arcuate nucleus; AgRP, agouti-related peptide; GCs, glucocorticoids; GR, glucocorticoid receptor; IR, insulin receptor; LepR, leptin receptor; MC4R, melanocortic 4 receptor; α-MSH, α melanocyte stimulating hormone; NPY, neuropeptide Y; POMC, pro-opiomelanocortin; PVN, paraventricular nucleus; Y1/Y5, Y1 and Y5 receptors; 3V, 3rd Ventricle.

The most studied anorexogenic neurons in the ARC are pro-opiomelanocortin (POMC) neurons as they synthesize POMC as well as cocaine and amphetamine regulated transcript peptides (CART), both of which decrease food intake. They are almost exclusively located in the ARC and project mainly to the PVN, lateral hypothalamus (LH) and the brainstem areas, which are all associated with control of energy balance (Figure 1).

POMC can be cleaved to numerous peptides including adrenocorticotrophic hormone (ACTH), β-endorphin and α-melanoctye stimulating hormone (α-MSH). The most widely studied anorexigenic neuropeptide in the ARC is α-MSH, which acts as an agonist at melanocortin 3 receptors (MC3R) and melanocortin 4 receptors (MC4R) in the hypothalamus. These receptors are highly expressed on second order neurons in the PVN and their activation leads to a reduction in food intake and an increase in energy utilization. Understanding the function of POMC has been enhanced by the use of knock-out models. The global POMC knock-out mouse has hyperphagia, leading to excessive body weight gain (Coll et al., 2005). Additionally, deletion of either the MC3R (Butler et al., 2001) or MC4R (Huszar et al., 1997) also leads to an obese phenotype with increased fat mass. Furthermore, genetic mutations in the POMC gene have been shown to cause obesity in patients (Krude et al., 1998; Challis et al., 2002; Farooqi et al., 2006; Creemers et al., 2008). Therefore, POMC derived peptides clearly have roles in reducing food intake to control body weight.

The other major anorexigenic neuropeptide in the ARC is CART, however, it is less widely studied than POMC, due to a lack of understanding of the receptors for this peptide (Lau and Herzog, 2014). CART is released from the same ARC neurons that release POMC and has a similar effect to reduce food intake and increase energy expenditure. But in contrast to POMC, CART is more widely expressed throughout the brain and the peripheral tissues (Douglass et al., 1995; Couceyro et al., 1997; Ekblad, 2006; Vrang, 2006; Kasacka et al., 2012). When CART knock-out mice are fed a high caloric diet, they demonstrate the expected increases in food intake and body weight (Asnicar et al., 2001; Wierup et al., 2005; Moffett et al., 2006), giving an insight into the role of CART in energy balance.

### Orexigenic ARC neurons

The orexigenic neurons in the ARC primarily release neuropeptide Y (NPY), agouti-related peptide (AgRP), and γ-aminobutyric acid (GABA) to increase food intake. Their cell bodies are located in the ARC and the neurons project to other hypothalamic regions including the PVN, DMH, perifornical area (Karatsoreos et al., 2010), LH, and the medial preoptic area (MPO) (Chronwall et al., 1985; Morris, 1989).

The role of these neurons in the control of food intake has been highlighted in a recent study where each of the individual components were deleted, demonstrating that NPY, AgRP, and GABA have a synergistic role in the control of food intake (Krashes et al., 2013). However, ablation of the whole orexigenic ARC neuron postnatally leads to starvation of the animal (Gropp et al., 2005; Luquet et al., 2005), indicating the essential role of these neurons in regulating appetite.

AgRP is an antagonist at the MC4R, blocking the effects of α-MSH in the energy regulating pathway and thereby increasing food intake. Interestingly, deletion of AgRP from the neuron can result in an unchanged (Corander et al., 2011) or a very mild phenotype, which is only seen in older mice (Wortley et al., 2005). These mice have a decrease in body weight from 6 months of age, associated with an increase in energy expenditure. Although, knocking out the peptide has such a small effect, administration of AgRP by intracerebroventricular (icv) injection increases food intake, which is sustained for up to a week (Rossi et al., 1998; Hagan et al., 2000). More recently, using genetic manipulation it was noted that AgRP has a role in prolonged, rather than short-term feeding (Krashes et al., 2013).

NPY binds to Y1 and Y5 receptors in the hypothalamus to exert its effects on food intake and energy balance (Morton and Schwartz, 2001). Its role in these processes has been clearly demonstrated in a well-designed study, where an icv injection of NPY induced feeding in rodents and which was maintained for a number of hours (Clark et al., 1984). Furthermore, repeated injections of NPY over several days resulted in increased food intake, body weight and adiposity (Stanley et al., 1986). Conversely, injecting an antisense oligonucleotide or antiserum to NPY decreased food intake and body weight gain in rats (Shibasaki et al., 1993; Hulsey et al., 1995). Curiously, global knock-out of NPY results in normal body weight and food intake as well as a normal response to a 48 h fast (Erickson et al., 1996). This must be a consequence of other pathways compensating in the absence of NPY.

As well as the two neuropeptides, the neurotransmitter GABA is also released from orexigenic ARC neurons. From studies using designer receptors exclusively activated by designer drug (DREADD) technology, it appears that GABA has a similar role to NPY, such that it can influence the initial phase of feeding and can act as a substitute for NPY (Krashes et al., 2013). Together, the three factors released from orexigenic ARC neurons promote an increase in food intake.

### Glucocorticoids and the regulation of food intake

Glucocorticoids (GCs) are steroid hormones secreted by the adrenal gland under the control of hypothalamic-pituitary-adrenal (HPA) axis and are known to have a role in the regulation of food intake as well as energy balance. They exert their effects by binding and activating the glucocorticoid receptor (GR). This triggers a cascade of events resulting in GR acting as a transcription factor and binding to glucocorticoid response elements (GREs) such as those present in the promoter regions of AgRP (Lee et al., 2013) and NPY (Misaki et al., 1992).

Models of GC excess in mice have highlighted their ability to increase food intake and body weight. Both active corticosterone (equivalent to cortisol in humans) and inactive 11-dehydrocorticosterone (cortisone in humans) can mediate these effects (Karatsoreos et al., 2010; Harno et al., 2013; Morgan et al., 2014). Furthermore, these models mimicked metabolic syndrome and Cushing's syndrome, both of which are associated with glucocorticoid excess and are characterized by hyperphagia. Similar clinical symptoms can be seen as a result of the adverse effects seen with administration of chronic synthetic “steroid” (glucocorticoid) treatment. In addition, adrenalectomy and removal of endogenous GCs leads to a reduction in body weight and food intake in genetically obese rodents (Marchington et al., 1983; Makimura et al., 2000).

GCs also have a role in altering the expression of both ARC orexigenic and anorexigenic neuropeptides. This has been explored by either depleting the levels of GCs or significantly increasing the amounts of GC exposure. AgRP is positively regulated by GCs in multiple studies (Savontaus et al., 2002; Coll et al., 2005; Kim et al., 2013; Lee et al., 2013). The results are less clear cut for NPY with studies demonstrating that dexamethasone, a synthetic GC, increases NPY expression (Goto et al., 2006; Yi et al., 2012), but corticosterone, the endogenous GC in rodents, has no effect (Coll et al., 2005). The control of POMC by GCs is also controversial with studies showing both positive (Savontaus et al., 2002; Uchoa et al., 2012) and negative (Beaulieu et al., 1988; Makimura et al., 2000; Gyengesi et al., 2010) regulation by GCs.

### Other peripheral hormones acting on neural networks

Other peripheral hormones, principally leptin and insulin, can cross the blood brain barrier to act in the hypothalamus on ARC orexigenic and anorexigenic neurons. Both of these peripheral hormones have receptors on both classes of neurons, allowing these factors to affect firing and signaling of the neuron and in turn have an effect on food intake and energy balance.

Leptin, released by adipocytes, circulates in direct proportion to the amount of adipose tissue (Considine et al., 1996). It is anorexigenic and some of its effects are mediated by POMC neurons as a result of increasing α-MSH secretion (Cowley et al., 2001; Munzberg et al., 2003; Guo et al., 2004). In addition, orexigenic AgRP/NPY neurons also express leptin receptors (Elmquist et al., 1998) and leptin reduces the expression of both AgRP and NPY neuropeptides (Stephens et al., 1995; Mizuno and Mobbs, 1999). Evidence for the functional role of leptin has come from studies where leptin has been administered centrally in rodents and decreases food intake and body weight (Campfield et al., 1995; Weigle et al., 1995). There are also studies in obese children with congenital leptin deficiency, where leptin replacement has been able to reduce body weight and hyperphagia (Farooqi et al., 1999). However, leptin is not a suitable treatment for most patients with obesity as they already have high circulating levels of leptin because they have developed leptin resistance, a condition where leptin is unable to induce its effect of lowering of food intake and therefore body weight (Schneeberger et al., 2014).

Insulin can act in the ARC to produce an anorexigenic effect, reducing food intake and body weight (Woods et al., 1979). Insulin receptors are widely expressed in the brain and co-localize with both POMC and AgRP neurons (Marks et al., 1990; Benoit et al., 2002). Activation of the insulin signaling pathway in the ARC decreases NPY and AgRP expression as well as increasing the expression of POMC to give the anorexigenic effect (Schwartz et al., 1992; Sipols et al., 1995; Benoit et al., 2002; Plum et al., 2006). As with leptin, there is evidence for central insulin resistance, decreasing the ability of insulin to reduce food intake in obese patients (Hallschmid et al., 2008).

Another peripheral hormone that plays an important role in the regulation of energy balance is the gut-derived orexigenic peptide hormone, ghrelin. It acts via the growth hormone secretagogue receptor (GHS-R) which is present at a high density in the hypothalamus, specifically in the nuclei involved in body weight and food intake regulation such as the ARC, ventromedial hypothalamus, DMH, and PVN (Mitchell et al., 2001; Zigman et al., 2006). The ghrelin-induced increase in food intake is mediated by the NPY/AgRP pathway as genetic ablation or pharmacological inactivation of AgRP and NPY blocks the orexigenic effects of ghrelin (Nakazato et al., 2001; Shintani et al., 2001; Chen et al., 2004). Moreover, central administration of ghrelin to rats leads to an increase in the mRNA expression of both NPY and AgRP (Kamegai et al., 2001; Nakazato et al., 2001; Shintani et al., 2001).

## Experimental evidence for the programming of appetite and obesity

There are many examples within the literature demonstrating a link between maternal nutritional insults and detrimental effects on the fetus. Both maternal under- and over-nutrition have been extensively described with additional factors such as the timing of the insult, the gender of the offspring and importantly, the severity of the nutritional change leading to complex outcomes. As a result, deciphering the pathways and the key genes affected is challenging. The hypothalamic appetite signaling pathway is imperative in these processes and is altered following perturbations in the maternal nutritional environment in fetal and postnatal offspring.

### Maternal undernutrition and its effects on the offspring

Maternal undernutrition is recognized as being a cause of fetal programming leading to the offspring having an increased propensity to develop hypertension, type 2 diabetes and cardiovascular disease (Barker, 2005). The Dutch famine (1944–1945) has been the basis for initial pioneering epidemiological studies demonstrating the negative associations between maternal environment and disease outcomes in the offspring (Ravelli et al., 1976). One of the main characteristics of maternally programmed offspring is altered birth weight, which was a marker that these studies initially utilized to identify programmed offspring. It was found that mothers exposed to famine during early gestation had offspring with normal birth weight but with an increased possibility of becoming obese, whereas mothers exposed to famine later in pregnancy had offspring with lower birth weights (Ravelli et al., 1976; Sharkey et al., 2009). These findings clearly show that exposing the fetus to maternal undernutrition during different times of fetal development can induce differential outcomes. Longitudinal analysis of this cohort revealed that at approximately 50 years of age the maternally-programmed offspring had compromised glucose tolerance and higher insulin concentrations (Ravelli et al., 1999; Mostyn and Symonds, 2009). Extensive analysis of these individuals has led to the understanding that these changes may be a consequence of impaired insulin secretion (de Rooij et al., 2006). However, the precise mechanisms governing these changes are difficult to elucidate due to the restrictions of using the human model. This is particularly true when trying to assess the hypothalamic regulation of energy balance. As a result, several animal models have been established to characterize the potential pathways involved.

#### Caloric restriction, intrauterine artery ligation, and twinning as models of adverse events in pregnancy

A range of programming models have been developed to mimic the human maternal undernutrition paradigm. These animal models are critical in defining the systems and underlying mechanisms that may be involved in the increased susceptibility of the children to develop disease in adulthood, following exposure to maternal nutritional programming. These models have included caloric restriction, intrauterine artery ligation, and twinning. Caloric restriction has been one of the most common paradigms, with studies reducing the calorie intake by 50–70% in the earlier studies (Delahaye et al., 2008; Breton et al., 2009) and then to more moderate levels in more recent studies (Stevens et al., 2010). Initially the high levels of caloric restriction were essential in defining changes that may be occurring in mothers exposed to famine. However, using moderate restriction could be more applicable to mimicking the effects of women dieting during the initial periods of conception to increase their chances of conceiving or because of social pressures to be thin. These studies have been vital in highlighting the effects of maternal undernutrition on hypothalamic mechanisms in the fetus. Nevertheless, the inconsistencies between the investigations about the severity and timing of the insult should be addressed.

One of the proposed mechanisms for maternal programming of the fetus is altered placental blood flow leading to the birth of small for gestational age babies (Sanders et al., 2004). Bilateral uterine ligation techniques have been developed as a tool for investigating the effects of placental insufficiency (Gagnon, 2003). Glucose is a necessary component of the maternal and fetal system, which the fetus will utilize to aid many developmental processes. Accordingly, any induced or natural decreases in blood flow can dramatically reduce the levels of glucose that the fetus is exposed to, leading to hypoglycaemia. This in turn can impact on insulin levels and pancreatic β-cell function. Consequently, the fetus must adapt to this situation by altering other metabolic parameters with expected adverse outcomes such as impaired glucose tolerance. However, there is evidence to suggest that this model of maternal programming and its outputs are difficult to reproduce (Neitzke et al., 2008). Therefore, models of bilateral uterine artery ligation require further development before significant conclusions can be made about any changes that are observed.

Over the years the number of mothers giving birth to twins has risen due to artificial reproductive technology and multiple ovulations, because mothers are having children later in life (Blondel et al., 2002; Ombelet et al., 2006; Muhlhausler et al., 2011). More recently, twins have been found to have some similarities with programmed offspring, having reduced birth weight, potentially due to the twins being exposed to a maternal environment where the fetuses must compete with each other for nutrition (Rumball et al., 2008). Additionally, twins have a reduced placenta size, as well as being in a restricted space. This may alter their developmental trajectory to provide compensatory mechanisms to ensure their survival. Studies have already found that these possible compensatory mechanisms can lead to altered glucose regulation and higher fat mass in adulthood (Poulsen et al., 1997; Ribel-Madsen et al., 2012). Despite these findings there are inconsistencies in the development of diseases in adult twins (Phillips et al., 2001; Muhlhausler et al., 2011). This may be because these investigations have omitted to use singletons as their control and therefore, it is difficult to ascertain if any changes are because of the inherited genotype of the animal or if they are caused by the maternal environment (Muhlhausler et al., 2011). Nevertheless, some studies have compared their data to effects seen in singletons. They have found changes similar to maternally undernourished animals with impaired glucose and insulin signaling and altered hypothalamic pathways (Rumball et al., 2008; Begum et al., 2012) indicating that these mechanisms are important in programming.

#### The effects of maternal undernutrition on the hypothalamic appetite signaling pathway

The development of a functional hypothalamus occurs in two phases, which involves neurogenesis and the formation of functional circuits (axon growth and synaptogenesis). In precocial species such as humans, sheep and non-human primates, the development of hypothalamic neural projections occurs at the fetal stage. However, in altricial rodents the hypothalamus is immature at birth and development continues during the first 2 weeks of postnatal life when neurons send axonal projections to their target sites and functional synapses are formed (Bouret, 2010). As a result the rodent hypothalamus is not fully formed until after weaning. Thus, any changes in the maternal nutritional environment during these critical periods will severely impact on this system potentially altering the offspring's developmental trajectory. Several studies on maternal perinatal undernutrition in rodent models have produced key evidence that these programmed offspring have low birth weight, exhibited hyperphagia in postnatal life and develop obesity, insulin resistance in adulthood (Vickers et al., 2000; Yura et al., 2005). This hyperphagia is exacerbated when the offsprings are fed high caloric diet in the post-weaning period (Vickers et al., 2000; Yura et al., 2005). Moreover, alterations in hypothalamic cell proliferation and impaired axonal elongation have been observed in the perinatally undernourished offspring (Delahaye et al., 2008; Breton et al., 2009; Garcia et al., 2010). These changes modify the density of the NPY and POMC neurons. This could have direct consequences on the animal's food intake and energy expenditure and depending on which neurons are affected it could increase food consumption and body weight. Supporting evidence for this was provided by a study which showed a greater level of food intake as a direct result of the maternal insult (Garcia et al., 2010).

In addition to structural variations in the hypothalamus, these models have also presented modifications in the expression of ARC neuropeptides that have a central role in the energy regulating pathway. This includes the anorexigenic POMC, and the orexigenic neuropeptides, AgRP and NPY. Despite clear differences in the severity and timing of the nutritional insult, the outcome of maternal undernourishment is consistent, with increased hypothalamic NPY and AgRP and decreased POMC gene expression in postnatal rodent offspring (Lopez et al., 2005; Delahaye et al., 2008; Breton et al., 2009; Cripps et al., 2009; Shin et al., 2012).

As the hypothalamus develops postnatally in rodents, animal models that specifically target the postnatal period have been developed. This has been done by adjusting the litter size in rats and mice. It has been found that pups raised in large litters (therefore less maternal milk) have attenuated pre-weaning growth. This was associated with an increased number of NPY neurons and higher NPY concentrations in the ARC (Plagemann et al., 1999c). These results show that postnatal undernutrition in rats leads to alterations in hypothalamic neural circuits regulating food intake and body weight.

Whilst the rodent model has been extremely useful for characterizing maternal programming paradigms, the length of gestation and the level of hypothalamic development during this time is not comparable to humans (Symonds and Budge, 2009; Bouret, 2010). As a result findings from species that are more developmentally similar to humans, such as sheep, are vital in enhancing our further understanding. These studies have shown that moderate levels of maternal undernutrition during early gestation led to increases in GR expression levels but no changes in POMC or NPY mRNA expression at the fetal stage (Stevens et al., 2010; Begum et al., 2013). However, evidence from higher mammals such as baboons have shown a decrease in POMC expression and increased NPY expression in the baboon fetus (Li et al., 2013). Therefore, changes may become apparent depending on the insult and the species investigated.

There is a general lack of studies analyzing adult offspring. This is problematic because the impact of programming on the susceptibility of the adult offspring to develop diseases is the key issue. A few longitudinal studies have been reported (Stevens et al., 2010; Begum et al., 2013). In these, it has been shown that maternal undernutrition leads to increases in GR expression levels which are sustained from fetal to adult life. There were also associated decreases in POMC expression in adulthood which may be a consequence of the change in GR (Stevens et al., 2010; Begum et al., 2013).

### The effects of maternal overnutrition on the hypothalamic energy regulating pathway

Over recent years the focus of research in the programming field has moved from studying the effects of maternal undernutrition to maternal overnutrition. Investigating this programming paradigm has become increasingly vital due to the rise in the levels of obesity within developed countries such as the USA and UK. Partly to blame for this surge in obesity is the increased consumption of high caloric foods by the mothers, combined with reduced levels of exercise. The number of epidemiological studies establishing a clear link between increased maternal body mass index and the offsprings' susceptibility to disease in adulthood are extensive (Oken et al., 2008; Drake and Reynolds, 2010; Reynolds et al., 2010). An interesting study by Patti (2013), found that mothers who underwent bariatric surgery had children with a decreased propensity to develop obesity compared to offspring born before the mother was subject to surgery. This further highlights the negative implications of maternal overnutrition for the offspring (Patti, 2013).

Once again rodent models have been essential in detailing the effects of maternal high fat diet on the offspring. These studies have not only described changes that occur during pregnancy, but also those that may take place during lactation (Guo and Jen, 1995; Samuelsson et al., 2008; Tamashiro et al., 2009). As expected, the outcome of these studies has been the development of obesity in the offspring accompanied by impaired insulin and glucose homeostasis, as well as many other outcomes such as cardiovascular diseases (Williams et al., 2014). However, unlike maternal undernutrition, the findings from the maternal overnutrition studies have been conflicting and therefore, more difficult to interpret. Whilst some studies are in agreement that there are impaired anorexigenic and orexigenic nerve fiber projections from the ARC to the PVN (Bouret et al., 2008; Chen et al., 2008; Kirk et al., 2009), the levels of neuropeptide expression are extremely diverse. For example, when the mothers were fed a high fat diet throughout gestation and lactation there was increased POMC mRNA and lower amounts of NPY expression in the programmed offspring (Chen et al., 2008). The rise in POMC may have occurred to provide some form of compensatory mechanism within these animals. In contrast, some studies have shown decreases in POMC and NPY (Morris and Chen, 2009), whilst another found increases in CART, NPY, and AgRP (Lopez et al., 2005). Although the standard animal model of maternal overnutrition during gestation and lactation, gives rise to adverse effects in the offspring, it does not explain which effects are caused by gestational and which by lactational exposure to maternal high fat feeding. Indeed, Vogt et al. (2014), have demonstrated the importance in the timing of the maternal insult and the effects that this can have on fetal outcome. In this study it was found that giving mothers a high fat diet during lactation induced changes in the POMC and AgRP nerve fiber projections in the hypothalamus (Vogt et al., 2014).

In the aforementioned study, one possible underlying mechanism suggested to induce modified fiber projections was altered insulin signaling (Vogt et al., 2014). Indeed, when the insulin receptor on POMC neurons was ablated in offspring from mothers fed a high fat diet, this led to a rescue in the previously distorted nerve projections. As a result, this study is the first to demonstrate a clear link between altered insulin signaling in the hypothalamus and perturbations in the hypothalamic energy regulating pathways in a maternally programmed paradigm. Since in humans, ARC neuronal projections arise during the third trimester, these results indicate that changes in maternal glucose metabolism during this critical window may have metabolic effects on the offspring throughout life. This investigation did not rule out the possibility of other mechanisms affecting the structure of the hypothalamus and other studies have suggested that changes in hypothalamic connections may also be associated with altered leptin resistance (Kirk et al., 2009). These findings have been essential in determining potential underlying reasons for the changes that are being observed in maternal programming paradigms. Nevertheless, there are still large gaps in our knowledge of how nutritional insults impact on the maternal and fetal interface such that they are affecting peripheral regulators to modify central pathways in a negative manner.

Another approach to studying postnatal overfeeding is by reducing the litter size. Raising pups in small litters post birth can potentially decrease competition for the mother's milk and therefore increase nutritional availability. As a result, there is a greater chance of the pups developing obesity, hyperphagia, and impaired glucose tolerance. Indeed, one study found that this led to increased numbers of neurons producing the orexigenic neuropeptide galanin in pups from reduced litters (Plagemann et al., 1999b). Taken together, these results demonstrate that exposure to prenatal or postnatal overnutrition in rodents results in substantial changes in the development of the hypothalamic architecture.

### Maternal gestational diabetes programs the hypothalamic neural network controlling appetite in offspring

Gestational diabetes is a common complication predisposing offspring to obesity and diabetes. The effect of maternal insulin deficiency on the offspring's hypothalamic appetite regulating system has been studied using rodents injected with the pancreatic islet toxin streptozotocin (STZ) during early pregnancy to develop a model of diabetes. The pups of diabetic mothers presented with impaired metabolic regulation including increased glucose and insulin levels, hyperphagia and increased body weight. In addition, they exhibited structural alterations affecting the density of AgRP and anorexigenic αMSH containing fibers in the PVN with altered leptin sensitivity (Steculorum and Bouret, 2011b). In a similar animal model, Plagemann et al. (1999a) has shown that offspring of diabetic dams have an increased number of galanin and NPY containing neurons (Plagemann et al., 1999a). Taken together, these results highlight that maternal gestational diabetes leads to malprogramming of hypothalamic orexigenic and anorexigenic systems in the offspring, contributing to metabolic disorders later in life. Perhaps more importantly, these acquired alterations in hypothalamic appetite related circuits can be prevented by normalizing gestational hyperglycaemia (Franke et al., 2005).

### Programming affecting leptin, insulin, ghrelin and glucocorticoids, and the impact on their role in the hypothalamus

As mentioned above, leptin can bind to ARC anorexigenic and orexigenic neurons influencing transcriptional activity. Increases in leptin levels are critical for the developing brain in assisting with increased fiber density and ensuring correct axonal projections from the ARC to other areas of the hypothalamus. Additionally, leptin is crucial for neuronal differentiation and migration (Bouret et al., 2004; Udagawa et al., 2007). As a result, it is clearly evident that this hypothalamic energy regulating pathway is susceptible to changes in leptin levels which could be impaired in offspring of programmed mothers. Indeed, studies outlined above found altered leptin levels contributing to increased food intake in the offspring (Delahaye et al., 2008; Garcia et al., 2010). However, the exact mechanisms leading to these changes are yet to be elucidated.

Many studies have shown impaired insulin regulation in fetuses from nutritionally programmed mothers (Snoeck et al., 1990; Chamson-Reig et al., 2006; Theys et al., 2009; Todd et al., 2009). It is understood that these changes are occurring because of modifications in the pancreas, such that there is deficient insulin production and secretion. Further evidence suggests that a maternal low protein diet can lead to pancreatic islets with deficient responses to glucose stimulation (Theys et al., 2009). There is also significantly reduced β-cell proliferation found in postnatal offspring (Chamson-Reig et al., 2006). In part these changes in the pancreas could be a compensatory mechanism developed by the fetus in response to hypoglycaemia experienced as a consequence of insufficient blood flow across the placenta. It is important to note that only one investigation has clearly described a direct link between peripheral insulin exerting its action on the insulin receptor in the hypothalamus and impaired POMC nerve fiber projections in the hypothalamus (Vogt et al., 2014).

Recently, ghrelin has been shown to play an important role in the normal development of ARC neural projections in neonatal mice and perturbations in its action during early life can affect leptin sensitivity and cause lifelong metabolic disturbances (Steculorum et al., 2015). Furthermore, acute ghrelin injection to mouse pups during second week of postnatal life lead to altered levels POMC and NPY mRNA (Steculorum and Bouret, 2011a). These results highlight the importance of the effect of ghrelin in influencing normal hypothalamic development. Moreover, ghrelin levels are altered by early life nutrition decreasing its ability to activate ARC neurons (Collden et al., 2015).

Additionally, although key regulators of the hypothalamic pathway such as leptin have been investigated in relation to maternal programming paradigms, very few studies have examined GCs. In particular, changes in the GR have been shown to influence the expression of neuropeptides and therefore, GR presents itself as an ideal candidate for further study. Indeed Li et al. (2013) and Begum et al. (2013) have demonstrated clear increases in hypothalamic GR expression associated with adverse outcomes within the offspring of maternally undernourished baboons and sheep, respectively. These studies highlight the need for more research to be carried out on the role of GR and other hypothalamic regulators, which the field currently lacks.

### Gender specific effects of maternal programming within the offspring

Whilst there has been much work carried out to determine the effects of maternal programming, some investigations have failed to take into account the possibility of gender specific differences in the offspring. The possibility of sexually dimorphic outcomes has been clearly highlighted in several recent studies (Todd et al., 2009; Matthews and Phillips, 2012; Begum et al., 2013). For example, in maternally undernourished offspring it was found that males had an increased propensity to reduced insulin levels and were more sensitive to changes in insulin (Todd et al., 2009). Evidence from the literature suggests that this may be caused by the male brain being more sensitive to the nutritional alterations that are associated with leptin and insulin resistance (Clegg et al., 2003). Furthermore, it was found that male offspring from undernourished ewes developed an increase in fat mass which was not observed in the females (Begum et al., 2013). It could be hypothesized that these changes may have occurred as a consequence of decreased central actions of insulin, leading to lower levels of POMC expression and reduced inhibition of food intake. Investigations detailing the effects of maternal programming on the hypothalamic energy regulating pathway have found associated changes in leptin and insulin levels (Delahaye et al., 2008; Vogt et al., 2014). These peptides can be regulated by the levels of estrogen, which could provide a compensatory mechanism in females to circumvent the effects of under- or over- nourishment. However, more work needs to be carried out in order to fully understand sexually dimorphic changes as a consequence of maternal programming.

## Molecular mechanisms underlying developmental programming of obesity

The literature clearly demonstrates that environmental insults during critical periods of fetal development and early postnatal life can influence energy expenditure pathways, with permanent consequences for obesity risk. One plausible explanation is that nutritional insults during early life may interfere with organogenesis and thus altering organ structure and function (Langley-Evans, 2009). Another mechanism to explain developmental programming is epigenetic modifications, which regulate gene expression patterns in a spatial and temporal manner. Epigenetic marks are established during fetal development and early postnatal life (Jaenisch and Bird, 2003). These marks exhibit developmental plasticity in response to the environment and are maintained throughout life. Therefore, perturbations to such processes represent a principal candidate mechanism by which early life nutritional insults can induce persistent phenotypic alterations in adulthood. Accordingly, there has been recent growing interest in the role of epigenetic dysregulation of appetite regulatory genes in metabolic disorders such as obesity (Gluckman et al., 2009; Stevens et al., 2011; Waterland, 2014).

### Epigenetic mechanisms

Epigenetics is the study of stable alterations in gene activity that occur without changes in the underlying DNA sequence. Epigenetic modifications play a significant role in regulating chromatin assembly, DNA replication and repair as well as gene expression during cell differentiation and development (Delage and Dashwood, 2008). The candidate epigenetic mechanisms include methylation of cytosines in CpG dinucleotides, posttranslational modifications of histone proteins and micro-RNA mediated gene regulation. Most importantly, these epigenetic modifications function synergistically to maintain chromatin conformations in a cell type specific manner to influence transcriptional activity. Up to now, most of the studies of early life influences on epigenetic regulation of genes have focused on DNA methylation and a few on histone modifications. Therefore, the following section will focus on describing these two mechanisms and the potential influence they may have on central genes in the metabolic pathways of the fetus that may be altered as a consequence of maternal programming.

#### Influence of DNA methylation

DNA methylation is the most prominent, well-understood, and highly stable epigenetic modification. It is involved in controlling gene transcription, parental imprinting, X-chromosome inactivation, and also in preventing retrotransposon activity and genomic instability (Jones, 2012). The majority of DNA methylation occurs at the C5 position of cytosine residues that are adjacent to a guanine residue, known as a CpG dinucleotide. DNA methylation is a covalent modification characterized by the addition of a methyl group to the cytosine residue of DNA. This is catalyzed by DNA methyltransferases (DNMTs) which use S-adenosylmethionine (SAM) as a cofactor and methyl donor. DNMT3a and DNMT3b are *de novo* methyltransferases which methylate cytosine residues at the previously unmethylated CpG sites during germ cell development and embryogenesis. DNMT1 is a maintenance methyltransferase which predominantly methylates hemimethylated CpG dinucleotides and copies the methylation pattern in the newly synthesized DNA during replication (Hermann et al., 2004).

DNA methylation can affect gene expression by two mechanisms: (1) by directly preventing the binding of transcription factors to their recognition sites on the DNA (Watt and Molloy, 1988), and (2) it can silence gene expression by promoting the binding of methyl-CpG-binding proteins (MBPs) which directly recognize methylated DNA and recruit transcriptional co-repressor complexes containing histone deacetylases (HDACs) and histone methyltransferases (Boyes and Bird, 1991; Jones et al., 1998; Nan et al., 1998). As a result, DNA methylation can effectively carry out its regulation of transcriptional activity. In mammals, 70–80% of CpG dinucleotides are methylated and the remaining unmethylated regions of the genome that have higher levels of guanine and cytosine content, are clustered together in CpG islands (Illingworth and Bird, 2009). There are CpG islands in promoter regions of 70% of mammalian genes and promoter CpG methylation is generally associated with gene silencing (Bird, 2002).

#### Transcriptional regulation by histone modifications

Histones are targets for epigenetic modifications which have profound effects on transcriptional activity, because of their role in packaging DNA into nuclear chromatin. The N-terminal region of core histones designated as H2A, H2B, H3, and H4 are subjected to numerous covalent modifications which influence both chromatin organization and gene expression. The posttranslational modifications of histones include acetylation, methylation, phosphorylation, ubiquitination, and sumoylation (Kouzarides, 2007). Acetylation of histones at specific lysine residues is a highly dynamic process regulated by two families of enzymes with opposing actions, histone acetyltransferases (HATs) and HDACs. HATs catalyze the transfer of acetyl groups to the lysine residues utilizing acetyl-CoA as cofactor whereas HDACs function to reverse acetylation (Sterner and Berger, 2000). Histone acetylation induces open chromatin conformation and correlates with transcriptional competence. Specifically, acetylation at histone 3 lysine 9 and 14 (H3K9/14ac) is associated with transcriptional initiation and H3K36ac is associated with transcriptional elongation. Histone methylation can be targeted at either lysine or arginine residues by lysine (HKMT) or arginine (PRMT) methyltransferases respectively. Histone arginine methylation is involved in transcriptional activity whereas lysine methylation has been associated with either activation or repression depending on the residue modified. For example, methylation of H3K4 is associated with transcriptional activation whereas methylation of H3K9 and K27 correlates with gene silencing (Kouzarides, 2007). Thus, combinations of different histone modifications can influence the regulation of the opening and closing of the chromatin resulting in activation or repression of a given gene. Many studies have used these patterns of histone modifications, termed the “histone code,” as a marker for epigenetic analysis within their models.

### Epigenetic programming of obesity: candidate gene studies vs. genome wide association studies

Given that epigenetic modifications are influenced by environmental perturbations, several groups have investigated the effect of maternal nutrition on the epigenetic changes of key hypothalamic genes regulating energy balance and thereby impacting on obesity in the offspring. These are discussed in the following sections of the review. A candidate gene approach has several strengths, narrowing the focus on the role of specific genes enabling direct phenotypic correlations to be made with increased statistical power. However, this approach does not further the understanding of the role of possible alterations in novel genes or pathways in the offspring, which ultimately shape developmental trajectories. This highlights the need for the genome-wide association study (GWAS) to unravel novel pathways involved in fetal programming of obesity. Accordingly, a few studies have focused on the impact of in utero environmental insults on the offspring's genome-wide epigenetic alterations (Altobelli et al., 2013; Crudo et al., 2013; Suter et al., 2014). Although these studies are instrumental in identifying novel genes and pathways associated with fetal programming, they have limitations such as low statistical power with multiple testing problems (Amos et al., 2011). Thus, future studies should combine genome-wide analysis with candidate-gene approaches, which are key to identifying the mechanisms involved in the increased risk of metabolic disorders in the offspring.

#### Epigenetic effects on hypothalamic POMC as a consequence of maternal programming

In addition to genetic polymorphisms in POMC (Krude et al., 1998; Challis et al., 2002; Farooqi et al., 2006; Creemers et al., 2008), epigenetic variations could contribute to an individual's risk of obesity (Kuehnen et al., 2012). The anorexigenic POMC is a likely target for epigenetic modifications by programming because there is good evidence for its regulation by promoter methylation (Newell-Price et al., 2001). The POMC gene has two CpG islands, one identified at the 5′-promoter region and one downstream around the intron 2 and exon 3 boundary (Newell-Price, 2003; Kuehnen et al., 2012). However, the POMC promoter regions necessary for the regulation of the POMC gene in the pituitary, and the hypothalamus, are different. In the hypothalamus its expression is regulated by two highly conserved distal neuronal enhancers, nPE1 and nPE2 located 10–12 kb upstream of the transcriptional start site (de Souza et al., 2005). Several studies have illustrated that an altered nutritional milieu perinatally can program epigenetic changes in the key regulator elements of the POMC promoter thereby affecting the appetite signaling pathway and inducing an obese phenotype in the offspring. For instance, maternal undernourishment during the periconception period in sheep leads to fetal hypothalamic POMC hypomethylation in a region that is closely related to the nPE1 and nPE2 POMC enhancer regions (Stevens et al., 2010, 2011). Another investigation by Plagemann et al. (2009) demonstrated that neonatal overnutrition in rats was associated with hypermethylation of CpG sites within the Sp1 binding site, which is required for the leptin and insulin mediated expression of POMC in the hypothalamus. These acquired methylation patterns were accompanied by a metabolic phenotype including obesity and increased leptin, insulin and glucose levels (Plagemann et al., 2009). This study clearly demonstrates epigenetic programming of POMC by early life nutrition and its effect on the risk of developing metabolic disorders. Additional investigations have shown hypermethylation of the POMC proximal promoter region in 28 day old postnatal mice reared by dams with dietary supplementation of conjugated linoleic acids (Zhang et al., 2014). This led to suppression of POMC expression and increased food intake contributing to catch-up growth and metabolic changes such as hyperglycaemia and insulin resistance (Zhang et al., 2014). These results show that changes in early life nutrition can elicit epigenetic and associated expression changes in hypothalamic POMC in the offspring.

In a clinical study, Kuehnen et al. (2012) have shown that hypermethylation of a few distinct CpG sites at the intron 2-exon 3 border (CpG island 2) of the POMC gene from peripheral blood cell (PBC) DNA was found in obese compared to normal weight children. This increased methylation was associated with reduced binding of the p300 histone acetyltransferase and decreased POMC gene expression in human PBCs. However, it is difficult to understand how epigenetic changes in the POMC gene in PBCs relates to changes in the gene in the hypothalamus as many epigenetic modifications are tissue specific and related to the function of the gene in one specific tissue. Although, it could be that the CpG island 2 of the human POMC gene is a metastable epiallele (Waterland, 2014).

All of these studies indicate that POMC is an emerging candidate for epigenetic modifications as a consequence of maternal programming. Despite this, more focused investigations need to be carried out delineating the full extent of any epigenetic modifications and the potential mechanisms that may be causing them. Some of these underlying mechanisms could be associated with altered dietary factors which act as cofactors of many epigenetic modifier enzymes such as DNMTs, HATs, and histone methyltransferases which are discussed in detail in the later section of the review. However, this hypothesis still requires further investigation.

#### Programming epigenetic changes in the hypothalamic GR

GR is a nuclear receptor that is important in many developmental and metabolic pathways. It is also involved in the hypothalamic control of energy homeostasis by regulating the expression of neuropeptides such as POMC, NPY, and AgRP as discussed above (Beaulieu et al., 1988; Misaki et al., 1992; Drouin et al., 1993; Wardlaw et al., 1998; Gyengesi et al., 2010; Lee et al., 2013). Thus, hypothalamic GR represents a prime candidate for fetal programming which induces alterations in energy balance, predisposing the offspring to metabolic disorders.

Evidence shows a striking link between effects of maternal care on the stress response and the epigenetic status of the GR gene in the offspring (reviewed in Zhang et al., 2013) indicating that the GR is a candidate for programming. In a well-established sheep model of undernutrition, it was shown that maternal undernourishment resulted in decreased GR promoter methylation, and alterations in histone modifications associated with increased GR mRNA expression in the fetal hypothalamic regions involved in energy balance (Stevens et al., 2010; Begum et al., 2012). Fascinatingly, these epigenetic alterations of GR persisted up to 5 years of age in adult offspring and were accompanied by decreased POMC mRNA expression. These results show the enduring nature of the impact on expression changes in the neuronal networks regulating food intake and energy expenditure (Begum et al., 2013). Interestingly, epigenetic changes in the GR gene as a result of altered maternal nutrition have been observed in other tissues such as liver and hippocampus. These results indicate programming effects on the methylation patterns of tissue-specific alternate first exons or promoters of the GR gene in the offspring (Lillycrop et al., 2005, 2007; Ke et al., 2011). Lillycrop et al. (2005) demonstrated that folic acid supplementation to a protein-restricted maternal diet prevented the epigenetic changes observed in the GR gene in the liver of the offspring. These results suggest the role of one-carbon metabolism in the epigenetic phenotype of the offspring (Lillycrop et al., 2005). Therefore, dietary alterations of these nutrients could provide a link between maternal diet and changes in the offspring's epigenetic signature.

#### Epigenetic alterations in other hypothalamic genes as a result of programming

Altered nutritional experiences during fetal or postnatal life impact on the epigenetic status of the other key hypothalamic genes involved in energy balance. For example, Mahmood et al. (2013) have shown that feeding new born rats with high-carbohydrate formula milk resulted in increased mRNA expression of NPY. This correlated with epigenetic alterations such as hypomethylation of specific CpG sites and increased H3K9 acetylation in the proximal promoter region of NPY and was associated with adult-onset obesity (Mahmood et al., 2013). Interestingly, overfeeding during neonatal life leads to hypermethylation in the promoter of the hypothalamic insulin receptor involved in the regulation of food intake and metabolism (Plagemann et al., 2010). Another study has shown that feeding mice with high fat diet during pregnancy and lactation resulted in global and promoter hypomethylation of hypothalamic dopamine and opioid-related genes in the offspring, leading to increased expression of these genes. These offspring showed more preference for palatable food rich in sucrose and fat (Vucetic et al., 2010). This suggests that dietary modulation during critical stages of the growth period can also affect epigenetic regulation of genes involved in food intake, body weight, and energy homeostasis thereby subsequent risk of developing obesity and related disorders.

### Mechanisms of changes induced in the epigenome as a result of programming

The precise mechanisms causing epigenetic changes induced by developmental programming is largely unknown. Altered levels of different nutrients, hormones or stress are likely to be the mediators of downstream signals of environmental insults. Micronutrients such as folate, choline, methionine, and vitamins are methyl group donors that contribute to the production of SAM through one-carbon metabolism. Since SAM is the universal methyl donor for both DNA and protein methyltransferases, variation in its levels can affect methylation patterns. For example, methyl donor supplementation to a high fat maternal diet prevented adverse effects such as body weight gain, behavioural changes and changes in DNA methylation and gene expression in the brain that were observed in the offspring of dams fed high fat diet (Carlin et al., 2013). Another way by which dietary factors can modify the epigenome is by modulating the expression or activity of epigenetic modifiers such as DNMTs. Accordingly, folic acid and other methyl group donors have also been shown to alter the expression levels of DNMT1, DNMT3a, and methyl CpG binding proteins involved in epigenetic gene silencing (Ghoshal et al., 2006; Lillycrop et al., 2007). The activities of other epigenetic enzymes such as HATs, HDACs, and demethylases are also susceptible to changes in the levels of cofactors such as acetyl-CoA, NAD, FAD, α-ketoglutarate and thus respond to altered nutrient intake (reviewed in Kaelin and McKnight, 2013). Additionally, evidence shows that both glucose and glucose metabolism induce genome-wide epigenetic alterations (Pirola et al., 2011). Therefore, it is conceivable that dietary factors can influence DNA methylation and histone modifications and thus affect signaling mechanisms regulating energy balance. However, despite this evidence, it is still not clear how fetal programming targets gene and tissue specific epigenetic modifications associated with metabolic disorders. It could be that the intensity and timing of the insult during early embryogenesis may impact the epigenetic alterations in the key metabolic genes in developing tissues. However, future studies are needed to understand the mechanisms by which the offspring's epigenomic configuration could be altered by maternal diet.

### Transgenerational effects on epigenetic programming of obesity

Evidence from human and animal studies indicate that developmental programming of obesity and other metabolic disorders is a transgenerational phenomenon and the transmission of effects to subsequent generations may occur even in the absence of exposure to adverse conditions (Aiken and Ozanne, 2014). Although mechanisms underlying transgenerational transmission of programming are not clear, evidence shows that it could be due to epigenetic inheritance via both maternal and paternal lineages (Vickers, 2014). The involvement of epigenetic mechanisms in transgenerational amplification of obesity was further confirmed when methyl donor supplementation (e.g., folic acid) during development prevented the increase in body weight in subsequent generations caused by maternal obesity (Waterland et al., 2008). Several studies using animal models have shown transgenerational effects of maternal under- or over-nutrition on glucose metabolism (Martin et al., 2000; Benyshek et al., 2004; Gniuli et al., 2008; Pinheiro et al., 2008; Jimenez-Chillaron et al., 2009) and cardiovascular and HPA axis function in F2 offspring (Bertram et al., 2008). However, the effect on appetite regulation in subsequent generations has not been widely studied and therefore requires further investigation. A complete review of transgenerational effects on epigenetic programming of obesity is outside the scope of this review. However, understanding the mechanisms of transgenerational inheritance is important for the development of intervention strategies to modulate the effects of developmental programming of offspring, grandoffspring and beyond.

## Conclusion

**Figure 2:**
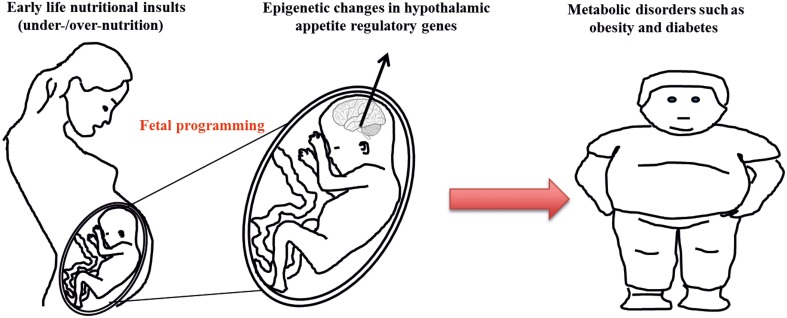
**Epigenetic programming of obesity**. Altered nutritional milieu at critical developmental period can program alterations in the epigenetic signature of hypothalamic appetite regulatory genes leading to changes in their gene expression pattern and increased susceptibility to metabolic disorders such as obesity and diabetes.

The compelling evidence reviewed here clearly shows that early life nutritional imbalance can induce persistent alterations, determining the permanent risk of obesity and other metabolic disorders in later life. Furthermore, epigenetic mechanisms might play an important role in these processes. Maternal under- and over-nutrition cause altered epigenetic regulation of genes that control food intake and energy expenditure in the hypothalamic neural network resulting in lifelong metabolic changes (Figure 2). This clearly suggests that the rise in the obesity epidemic could be at least in part explained by early life nutritional imbalances. The timing and the intensity of such events also seem to have a significant role to play in this process. This has led to the hypothesis that “we are what we eat and also what our mothers ate.” Our understanding of how environmental stimuli such as nutrition can induce epigenetic alterations is far from complete and further work is required in this area. However, given that there is clear evidence that these epigenetic modifications are reversible, there are possibilities that intervention strategies could combat the rise in obesity.

### Conflict of interest statement

The authors declare that the research was conducted in the absence of any commercial or financial relationships that could be construed as a potential conflict of interest.
